# The Liability of Hospital Trustees

**Published:** 1899-01-28

**Authors:** 


### THE LIABILITY OF HOSPITAL TRUSTEES.

We much regret that, through a misunderstanding, the name of the hospital and also of our informant, Mr. Sydney Holland, was inadvertently printed in connection with the correspondence which we published last week under this heading on p. 284.

